# *Septin14*, a gene specifically expressed in the testis and seminal vesicle of the Banna mini-pig inbred line (BMI)

**DOI:** 10.1590/1984-3143-AR2020-0521

**Published:** 2021-02-16

**Authors:** Pei Wang, Xia Zhang, Hailong Huo, Shuyan Wang, Xue Song, Jinlong Huo

**Affiliations:** 1 Faculty of Animal Science and Technology, Yunnan Agricultural University, Kunming, Yunnan, China; 2 Teaching Affairs Department, Yunnan Vocational and Technical College of Agriculture, Kunming, Yunnan, China

**Keywords:** Septin14, pigs, molecular cloning, subcellular localization, gene expression, protein purification

## Abstract

Septin14 is an important spermatogenesis related gene involved in the pathogenesis of male infertility that has not been well studied. Here, full-length Septin14 cDNA of the Banna mini-pig inbred line (BMI) was cloned using the RACE method and expressed in pig kidney epithelial cells (PK15) and *E. coli* Rosetta (DE3) cells. Septin14 expression was identified in somatic tissues and testis in different developmental stages. The pig Septin14 CDS is 1,299 bp long, and encodes a peptide (or protein) of 432 amino acids (MW=50.4 kDa). Phylogenetic analysis indicated that pig Septin14 was highly evolutionarily conserved. Subcellular localization of GFP-tagged Septin14 fusion protein revealed that Septin14 was distributed throughout the testicular cells. Among 34 pig tissues, Septin14 mRNA was found specifically in testis and seminal vesicle. In six different postnatal developmental stages, the testicular level of Septin14 mRNA was barely detectable on day 2, while the highest level occurred on day 75. The spatiotemporal expression profile of Septin14, reported herein for the first time in pig, indicated that Septin14 might be involved in the division, development and apoptosis of germ cells. Furthermore, using a pET prokaryotic expression system, we expressed and isolated recombinant 67.9 kDa Septin14 protein.

## Introduction

Septins are guanosine-5-triphosphate (GTP) binding proteins first described in yeast that can self-assemble into polymers and nonpolar oligomers (Mostowy and Cossart, 2012). Similar to microtubules and intermediate filaments, septins form a network of filamentous polymers and act as scaffolds for signaling proteins (Spiliotis and Dolat, 2016). Abnormalities in septin mutations and expression have been correlated with a number of diseases such as hereditary neuralgic amyotrophy and male infertility, Parkinson’s and Alzheimer’s diseases, as well as various solid tumors of epithelial origin (Kinoshita et al., 1998; Kuhlenbäumer et al., 2005; Peterson and Petty, 2010; Kuo et al., 2012; Dolat et al., 2014).

To date, 14 *Septin* genes have been identified in humans (Hall and Russell, 2004). Septin14 was first identified in human testis in 2007 (Peterson et al., 2007), and it is highly expressed in testis and is involved in spermatogenesis and cortical neuronal migration (Shinoda et al., 2010). Recent studies reported that the testicular tissues of men with hypo spermatogenesis, maturation arrest and sertoli cells had lower levels of Septin14 transcripts than normal men (Shafipour et al., 2014; Barzi et al., 2014), indicating that the Septin14 expression level was critical for human spermatogenesis. The Septin14 genes from *Homo sapiens* and *Mus musculus* have been extensively characterized (Peterson et al., 2007; Shinoda et al., 2010). However, *Sus scrofa domesticus* Septin14 has not been studied.

In 1980, we began breeding research of the Banna mini-pig inbred line (BMI). The foundation stock was selected from a sow and her litter, which has been closely reproduced and has an inbred property to some extent. The aim of this work was to clone and characterize the full-length pig Septin14 cDNA and investigate its subcellular localization and temporal and tissue expression profiles. Furthermore, we used a prokaryotic expression system to express BMI Septin14 and produced large quantities of the protein for further studies of the possible role of Septin14 in the pathogenesis of pig diseases.

## Materials and methods

### Pigs and sample collection

Thirty-four different organ samples from three 10-month-old BMI boars were used for spatial expression analysis, including brain, hindbrain, hypothalamus, brain axis, spinal marrow, pituitary gland, heart, liver, spleen, lungs, kidney, skin, muscle, duodenum, jejunum, ileum, colon, caecum, rectum, stomach, pancreas, esophagus, lymph gland, testis, epididymis, submaxillary gland, thyroid, adrenal, sublingual gland, thymus, pineal gland, bulbourethral gland, seminal vesicle and prostate. Animal care and experimental procedures of BMI in this study were in strict accordance with the Administration Regulations of Experimental Animals promulgated by the Ministry of Science and Technology of the People’s Republic of China. This study was approved by the Animal Care Committee of Yunnan Agricultural University.

The testes from postnatal day 2 (D2), D75, month 5 (M5), M10, M15, and year 3 (Y3) were used for temporal expression analysis (three samples in each stage). After collection, all samples were quickly frozen in liquid nitrogen and then transferred to -80 °C.

### Cloning of the pig *Septin14* gene

Total RNA from pig tissues was obtained using the RNAiso kit (TaKaRa, China). cDNA templates were generated from total RNA using the Invitrogen M-MLV reverse transcription kit. Based on the public database (HTGS), a *Septin*14-CDS-F/R primer pair was designed to amplify the full-length cDNA of pig Septin14, which was then sequenced. The remaining Septin14 primer sequences were designed based on this Septin14 sequence obtained (Table 1).

**Table 1:** Primers.

**Name**	**Primer sequence (5'-3')**	**Product size(bp)**
Primers for CDS		
Septin14-CDS-F	TCTGACATGGCAGAAAAACCA	1305
Septin14-CDS-R	TTATTTCTTACGATCTTTGTCCTT	
Primers for 5’RACE		
Septin14-GSP	AGATTTGTTCACCAACTGATGAGGC	
Septin14-NGSP	GACATACGCATTGGTTTTTCTGC	
UPM(F)	CTAATACGACTCACTATAGGGC	
NUP(F)	AAGCAGTGGTATCAACGCAGAGT	
Primers for subcellular location		
Septin14-C-F	GAATTCTGACATGGCAGAAAAACCA(*Eco*R I)	1315
Septin14-C-R	GGATCCTTATTTCTTACGATCTTTGTCCTT(*Xho* I)	
Primers for semi-quantitative RT-PCR		
18S rRNA-F	GGACATCTAAGGGCATCACAG	145
18S rRNA-R	AATTCCGATAACGAACGAGAC	
Primers for qPCR		
GAPDH-F	CCTTCATTGACCTCCACTACATGGT	183
GAPDH-R	CCACAACATACGTAGCACCAGCATC	
Septin14-qPCR-F	CAGATGATGAAGCTACCGTT	250
Septin14-qPCR-R	ACCTATACCGTTCATAGTGC	
Primers for prokaryotic expression		
Septin14-P-F	CCATGGCTGACATGGCAGAAAAACCA(*Nco* I)	1316
Septin14-P-R	GGATCCTTATTTCTTACGATCTTTGTCC(*Xho* I)	

Note: nucleotides underlined are restriction enzyme sites.

For 5’RACE and 3’RACE, 1 µg RNA extracted from adult pig testis was reverse-transcribed for cDNA according to the SMART^TM^ RACE Kit (Clontech Inc., USA). Primers (GSP and NGSP) were designed against SMART II oligonucleotide and NUP from this kit according to the sequenced pig Septin14 cDNA. The first PCR was carried out with a touchdown program. The template used in the second PCR was 20-fold more diluted than that in the first PCR. The second round PCR products were purified and cloned with the pMD-18T vector (TaKaRa, China) for sequencing.

### Sequence analysis

Septin14 amino acid sequences were aligned using Lasergene (DNAStar Inc., USA). The molecular weight (MW) and isoelectric point (pI) of the Septein14 proteins were calculated using ProtParam. Gene structure, subcellular localization, phosphorylation and glycosylation sites and conserved domains were predicted as described by Huo et al. (2015). Protein sequences similar to Septin14 were searched using the NCBI BLAST program. Multiple sequence alignments of Septin14 proteins of BMI with other known species were analyzed using DNAMAN. The multi-species phylogenetic tree was constructed using MEGA 7.0.

### Subcellular localization analysis

The eukaryotic gene expression plasmid pEGFP-C1-Septin14 was constructed from the pEGFP-C1 (Clontech inc., USA) plasmid digested using *Bam*HI and *Eco*RI restriction enzymes. Primers Septin14-C-F including start codon and Septin14-C-R including termination codon (Table 1) were used to amplify the Septin14 ORF.

Pig kidney epithelial cells (PK15) were transfected with pEGFP-C1-Septin14 until the cells reached 60-70% confluence. Briefly, the pEGFP-C1-Septin14 plasmid and lipofectamine 2000 were co-transfected into pig PK15 cells. After incubation in the dark for 24 h in a CO_2_ incubator, the PK15 cells were stained with Hoechst 33342 (Invitrogen USA), then washed with phosphate-buffered saline and viewed with an inverted fluorescence microscope (Zeiss, Germany) equipped with an AxioCam MRc5 color camera. Images were collected and processed using ZEN software (Zeiss, Germany).

### *Septin14* mRNA levels in various tissues

Total RNA (3 μg) from different tissues of pig was generated into cDNA. The reactions and protocols of semi-quantitative RT-PCR were similar to those for isolating Septin14 cDNA mentioned above. Septin14-CDS-F/R and 18S rRNA-F/R were used to amplify Septin14 and *18S rRNA* internal control, respectively.

### *Septin14* mRNA levels in testis tissues at different developmental stages

qPCR was used to quantify the temporal expression of Septin14 in testes from six postnatal periods of BMI boars using SYBR Premix Ex Taq (TaKaRa, China). *GAPDH* was amplified with primer pair GAPDH-F/R (table 1) as an internal control. The relative mRNA abundance of Septin14 was calculated using the 2^-ΔΔCt^ method. The values were relative to that of D2, which was used as a calibrator and normalized as 1. Statistical analysis was performed with the SPSS 20.0.

### Cloning the pig *Septin14* gene into prokaryotic expression vector pET32a

The entire ORF of the pig Septin14 gene was amplified using the specific primers Septin14-P-F/R (Table 1) and connected with *Eco*RI -*Nco*I sites of pET32a (+) vector (Novagen, Germany) with Trx and His-tag. Positive clones were confirmed by bacterial liquid PCR, restriction digestion and sequencing successively. The correct fusion expression vector was named pET32a(+)-Septin14.

### Purification and identification of Septin14 protein

The pET32a(+)-Septin14 was used to transform *E. coli* Rosetta (DE3) cells. A single transformed colony was picked and cultured overnight. The cultures were diluted by 50-fold into LB media and grew under vigorous shaking until the absorbance at 595 nm was in the range from 0.5 to 1.0. IPTG was then added to 0.5 mM and cultured for 2.5 h and 5 h at 37 °C.

The bacteria were collected with centrifugation, dissolved in BugBuster protein extraction reagent (Novagen, Germany) and lysed by sonication. The clear soluble supernatant and remaining insoluble pellet were isolated using 15% SDS-PAGE and then stained with Coomassie Brilliant Blue. His-Septin14 protein was purified with the prepacked Ni-IDA column tube (TransGen Biotech, China). The purified target protein was then isolated using 15% SDS-PAGE and verified by western blot.

## Results

### Cloning and sequence analysis of the pig *Septin14* gene

**Figure 1:**
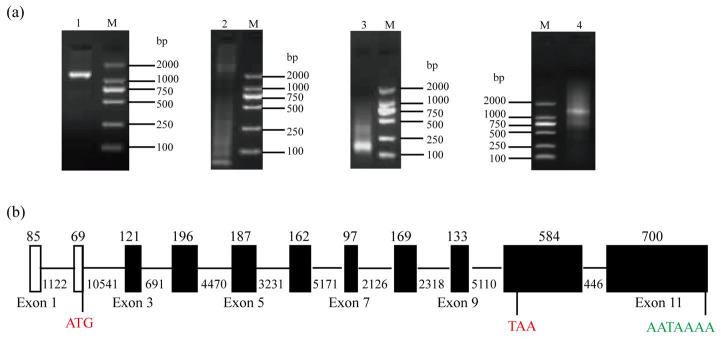
(a) PCR results for BMI Septin14 gene. Lane 1: RT-PCR product; lane 2: First round 5’RACE product performed with the forward primer UPM and the reverse primers Septin14-GSP1; lane 3: Second round 5’RACE product performed with the forward primer NUP and the reverse primers Septin14-NGSP1; lane 4: Second round 3’RACE product performed with the forward primer NUP and the reverse primers Septin14-NGSP2; lane M: DL2000 DNA Marker; (b) The exonic structure of BMI Septin14 gene. The black bars and lines denote exons and introns, respectively. Only the exons are draw to scale.

Using RT-PCR with Septin14-CDS-F/R as primers, a 1,305 bp partial cDNA fragment was obtained (Figure 1a). According to this partial cDNA sequence, 5’-end and 3’-end Septin14 cDNA was amplified using 5’RACE and 3’RACE. In the first round, a DNA smear appears with GSP primers (Figure 1a). In the second round, a 157 bp 5’-end 1,356 bp 3’-end sequence was obtained with NGSP primers (Figure 1a).

Gene structure analysis revealed that the pig Septin14 gene is located on the “+” strand of *Sus scrofa* chromosome 3 and spanned 37,716 bp from 16,989,788 to 17,027,503 (Sscrofa 11.1). This gene includes 11 exons of 85 bp, 69 bp, 121 bp, 196 bp, 187 bp, 162 bp, 97 bp, 169 bp, 133 bp, 584 bp and 700 bp in length (Figure 1b). The -GT and AG- sequences positioned at the end of every intron indicated that the Septin14 sequence follow the GT-AG boundary rule.

**Figure 2:**
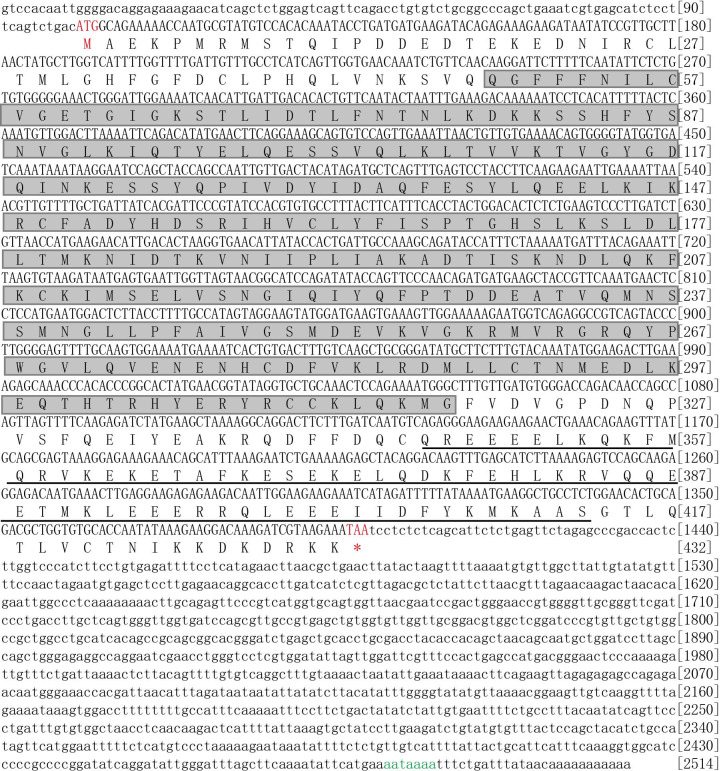
Nucleotide and corresponding translated amino acid sequences of BMI Septin14*.* The start codon (ATG) is colored in red and the stop codon (TAA) is annotated with an asterisk and also colored in red. The two conserved domain CDC_septin domain (49-317 AA) and the C-terminal coiled coil domain (346-413 AA) were boxed and underlined, respectively. The poly(A) signals (aataaaa) is colored in green.

We then assembled the 2,514 bp pig Septin14 full-length cDNA using DNAMAN software and submitted it to GenBank (accession number KU358532). There was a 100 bp 5’UTR and 1,115 bp 3’UTR and a 1,299 bp ORF sequence, which was deduced to encode a protein of 432 amino acids (Figure 2). At the end of the sequence, there was an 11 bp polyA tail and a typical polyA acidification signal (AATAAAA) was localized 15 bp upstream of the poly(A) tail (Figure 2). The calculated molecular mass and predicted pI of the theoretical polypeptide were 50.4 kDa and 7.2, respectively. A CDC_septin domain from the 49^th^ to 317^th^ amino acids and the C-terminal coiled coil domain (346-413 amino acids) were predicted by Protein BLAST alignment (Figure 2).

### Homology and phylogenetic analysis of pig Septin14 protein

Amino acid sequences of the Septin14 protein from *Camelus dromedaries* (XP_010979111), *Vicugna pacos* (XP_006201398), *Equus asinus* (XP_014697746), *Equus caballus* (XP_001493173), *Homo sapiens* (NP_997249), *Macaca fascicularis* (XP_005549591), *Macaca mulatta* (XP_014988929), *Ovis aries* (XP_004020983), *Bubalus bubalis* (XP_006070427), *Bos taurus* (NP_001095557), *Mus musculus* (NP_083102) and *Rattus norvegicus* (NP_001131142) were aligned with our cloned pig Septin14 (ANH21165). We found that BMI pig shares 91.7, 91.2, 84.3, 85.0, 82.6, 82.9, 82.9, 84.3, 85.0, 84.7, 79.7 and 77.5% sequence similarity with the Septin14 in the above vertebrates, respectively.

**Figure 3:**
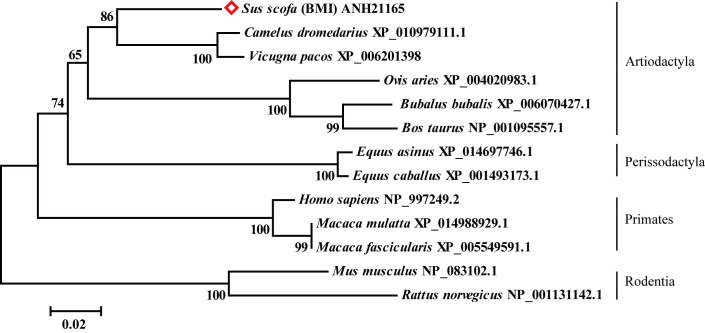
Septin14 amino acids phylogenetic tree of BMI with other 12 species.

A multi-species phylogenetic tree divided the 13 sequences of Septin14 into four main groups: Artiodactyla, Perissodactyla, Primates and Rodentia (Figure 3). Pig Septin14 was classified into the Artiodactyla group. It was most closely clustered with Septin14 from *C. dromedaries* and *V. pacos* indicating that they derived from a common ancestral gene. They then robustly clustered together with those from *O. aries*, *B. bubalis*, and *B. taurus* at 65% support.

### Subcellular localization of Septin14

**Figure 4:**
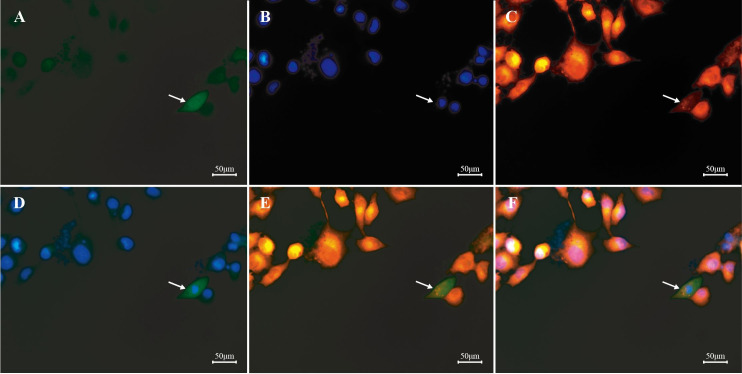
Subcellular localization of pig Septin14 protein in PK15 cells. Septin14 expression (green) was observed using fluorescence microscopy. Nuclei were observed by Hoechst 33342 staining (blue). (A) green fluorescent protein; (B) nucleus stained by Hoechst33342; (C) mitochondria stained by Mito Tracker; (D) superposition of GFP and nucleus; (E) superposition of GFP and mitochondria; (F) superposition of GFP, nucleus and mitochondria.

To determine Septin14 subcellular localization patterns, the fusion expression vector pEGFP-C1-Septin14 was constructed and transfected into PK15 cells using liposome 2000 transfection. Septin14 fusion proteins with GFP fluorescence signals were detected throughout the pig PK15 cells (Figure 4).

### Spatial expression of pig *Septin14* gene

**Figure 5:**
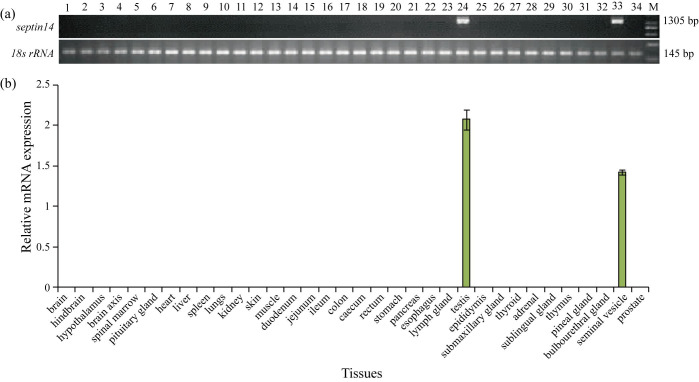
Tissue expression profiles of Septin14 gene in 34 tissues of BMI adult pigs by RT-PCR. *18S rRNA* was used for internal control. The vertical axis is gene relative expression quantification, and the horizontal axis is 34 tissues. Note: (a) M: DL2000 DNA Marker.

cDNA was synthesized from 29 non-reproductive organs and five reproductive organs of adult BMI pigs. Only the testes and seminal vesicle expressed Septin14 transcript based on RT-PCR (Figure 5), which implied that Septin14 is selectively expressed in testis to support normal testicular and seminal vesicle function.

### Temporal expression of pig *Septin14* gene

**Figure 6:**
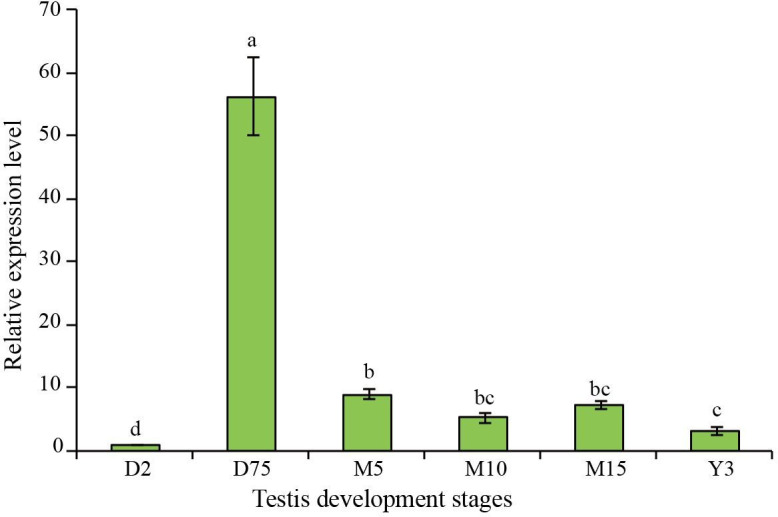
Expression analysis of the Septin14 gene in the testes of six development stages of BMI. *GAPDH* was used to normalize the expression level with 2^-ΔΔCt^ value. Note: D, days; M, months; Y, years.

Temporal expression of Septin14 in BMI boars was determined by qPCR. The expression levels of Septin14 at different stages of development from D2 to Y3 of age are summarized in Figure 6. At D2, Septin14 was expressed in testes at the lowest level, which was hardly detectable. At D75, Septin14 was significantly (P<0.05) up-regulated to the highest level, and was then maintained at a moderate to low level from M5 to M15, finally decreased to a low level at Y3.

### Expression of the His-tagged Septin14 protein

**Figure 7:**
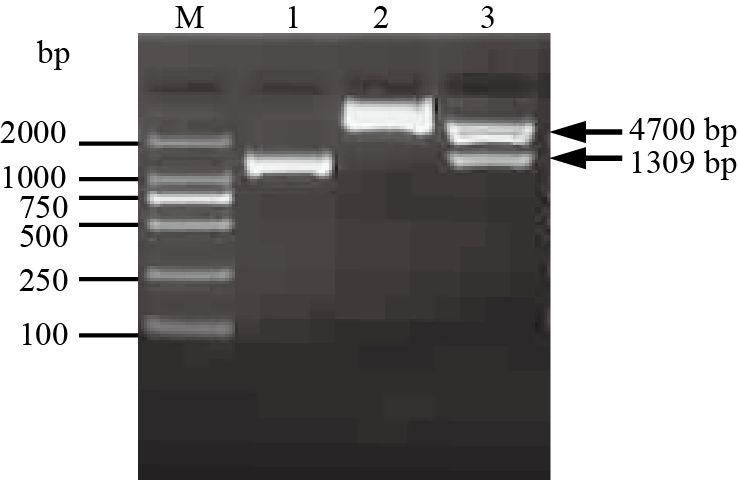
Construction of the recombinant expression plasmid pET32a(+)-Septin14. Note: lane 1: RT-PCR product of Septin14; lane 2: PCR product of Septin14 gene in bacterial liquid; lane 3: PCR product of Septin14 gene in plasmid; lane 4: pET32a(+)-Septin14 plasmid; lane 5: the recombinant pET32a(+)-Septin14 vector was digested by *Nco* I and *Xho* I; lane M: DL2000 DNA Marker.

We used RT-PCR to amplify the 1,316 bp full-length Septin14 gene sequence from BMI testis. The Septin14 sequence was inserted into pET32a(+) between the *Nco*I and *Xho*I sites to make the plasmid pET32a(+)-Septin14, which was validated by PCR, restriction enzyme digestion and DNA sequencing (Figure 7). The pET32a(+)-Septin14 plasmid encodes Septin14 fused to an N-terminal 6-His tag as part of a tandem Trx-His-S tag that facilitates purification of the fusion protein.

**Figure 8:**
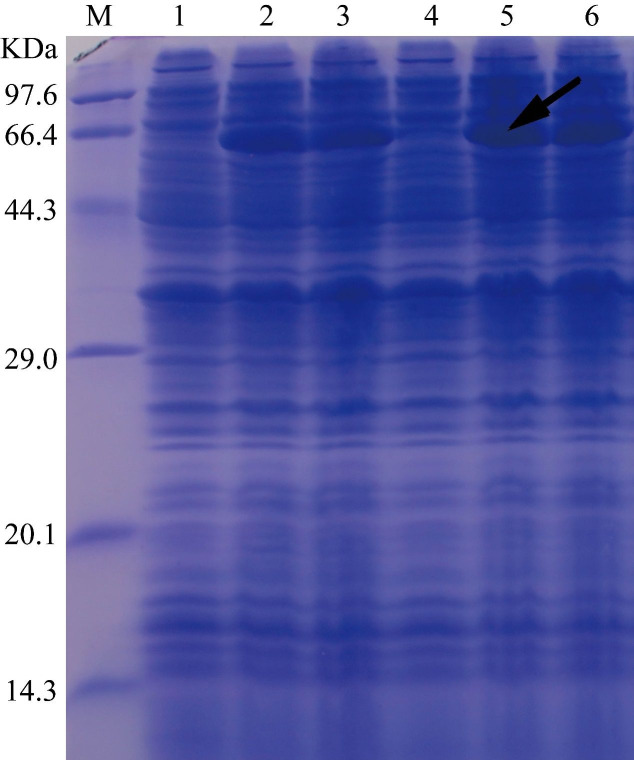
SDS-PAGE result of bacterially Septin14 expressed. The bacteria harboring the recombinant plasmid encoding the Septin14 vector were induced using 0.5 mM IPTG and the bacterial liquid was run by SDS-PAGE. Note: lane M: premixed marker (TaKaRa, China); lanes 1 to 3: protein at 0, 3.5, and 7 h after IPTG inducing at 37 °C, respectively; lanes 4 to 6: protein at 0, 8, 16 h after IPTG inducing at 20 °C, respectively. The arrow indicates the position of the fusion protein.

**Figure 9:**
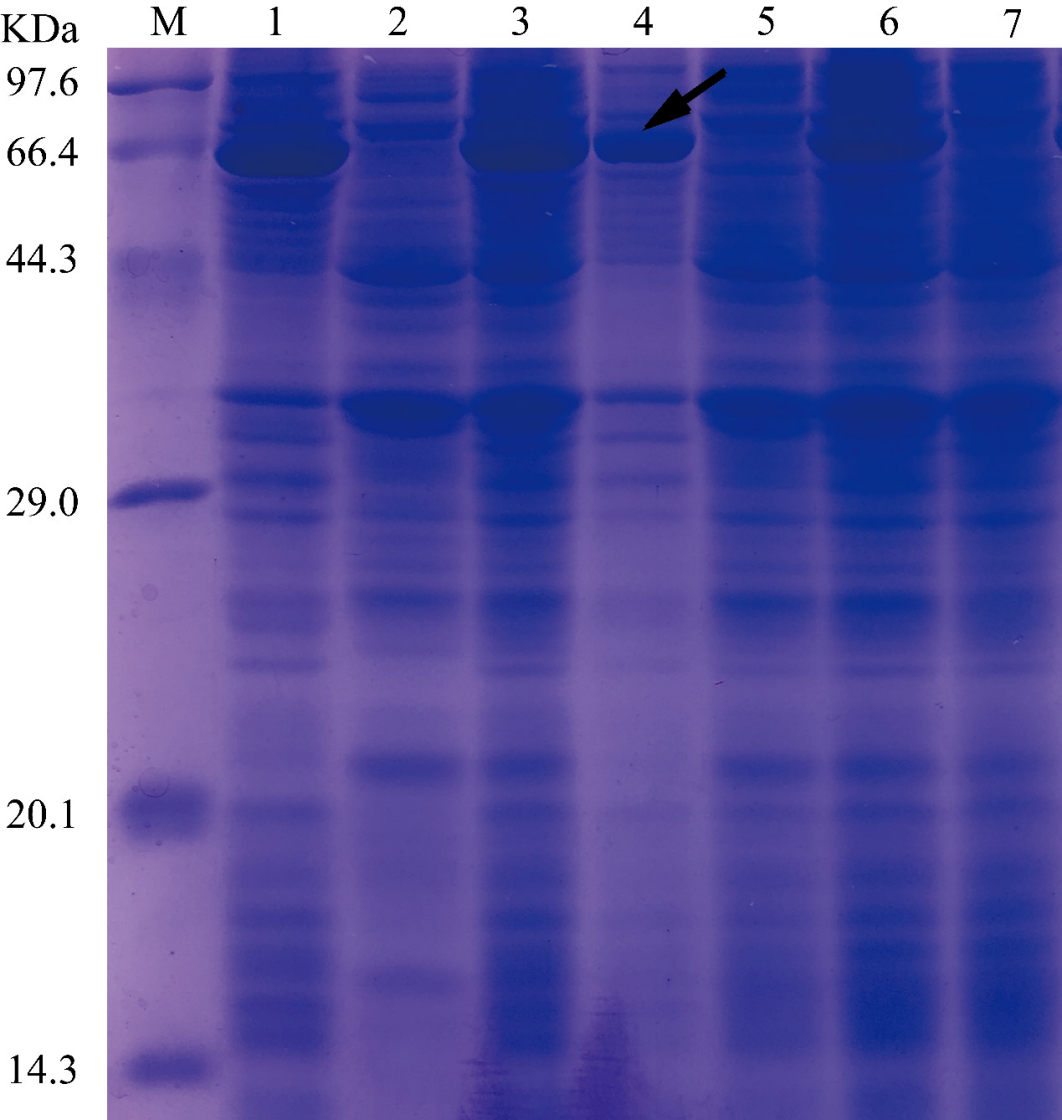
Solubility testing of the recombinant Septin14 protein. Note: lane M: premixed protein marker (TaKaRa, China); lane 1 and 4: insoluble inclusion bodies; lane 2 and 5: soluble supernatant; lane 3 and 6: proein after inducing by IPTG; lane 7: uninduced protein. The position of arrow is the fusion protein.

The pET32a(+)-Septin14 was transformed into Rosetta (DE3) cells. DE3 cells harboring pET32a(+)-Septin14 were grown at 37 °C or 20 °C. IPTG was added at 0.5 mM to the cell culture to induce the expression of His-tagged Septin14, which was subjected to analysis by SDS-PAGE. We observed an abundant protein of around 67.9 kDa from cells treated with IPTG, but not from control cells (Figure 8). Since the predicted mass of Septin14 is 50.4 kDa, and that of the Trx-His-S tag is 14.2 (11.8+0.8+1.6) kDa, the Septin14 fusion protein was predicted to be 64.6 kDa, which is close to the size of the protein from IPTG treated cells (Figure 8). We found that the recombinant Septin14 protein was present mainly in inclusion bodies and became soluble only after sonication (Figure 9).

### Purification and confirmation of recombinant Septin14 protein

**Figure 10:**
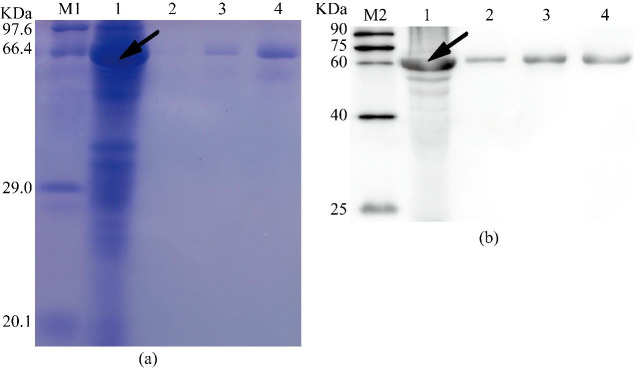
(a) Affinity chromatographic purification; (b) Western blot identification of the Septin14 fusion protein. Note: lane M1: premixed protein marker (TaKaRa, China); lane 1: insoluble inclusion bodies; lane 2 to 4: the target proteins were eluted by different concentration of imidazole elution buffer (100, 200, 300mM, respectively); lane M2: easy see Western marker (TransGen Biotech, China). the position of arrow is the fusion protein.

Given that the His6-tagged recombinant Septin14 protein accumulated in inclusion bodies, we used the Ni-Denature-urea buffer containing 8 M urea to solubilize His-Septin14 before purifying it using Ni^2+^-affinity chromatography. To determine the best imidazole concentration of eluent, a series of imidazole concentrations (from 0.1 to 0.3 M) was used to elute His-Septin14. We found that His-Septin14 was mainly eluted when the elution buffer contained 0.3 M imidazole. SDS-PAGE analysis indicated a 67.9 kDa size (Figure 10a) which reacted with a monoclonal anti-His antibody (Figure 10b). Therefore, we have purified the His6-Septin14 protein with a size of 67.9 kDa.

## Discussion

Septins are a group of GTP binding proteins found primarily in eukaryotes from fungi to animals, as well as some green algae (Weirich et al., 2008; Douglas et al., 2005). Different septins interact with each other to form septin complexes that can further organize into filaments, rings and gauzes. Septin structures provide a scaffold for protein binding or hinder the diffusion of molecules among compartments in the cell, thereby contributing to appropriate protein localization/distribution. (Mostowy and Cossart, 2012; Kinoshita, 2006). In humans, 14 septin genes encode dozens of polypeptides, many of which comprise heterooligomeric complexes. They play different functions in the cell including polarity determination, cytoskeletal reorganization, vesicle trafficking, mitosis, membrane dynamics, and exocytosis (Ihara et al., 2005).

Septin14 was discovered by Peterson and colleagues in 2007 when they were working on *septin9* as a potential candidate of genes involved in mammal spermatogenesis and male infertility (Peterson et al., 2007). We isolated cDNA of the pig Septin14 gene and examined its transcription in different tissues at various stages of development. Moreover, we also expressed a His6-tagged recombinant Septin14 protein in *E. coli* and purified it.

The pig Septin14 is a neutral protein with 50.4 kDa molecular weight, which has higher homology with *Camelus dromedaries* and *Vicugna pacos* Septin14 than with those of other species. This result indicated that any functional differences of pig Septin14 from those of *C. dromedaries* and *V. pacos* are likely minor. Therefore, studies of pig Septin14 could be a reference for us to understand some functions of Septin14 in these two species.

Two conserved structural domains, including a CDC_septin domain and the C-terminal coiled coil domain were found in pig Septin14 protein. These results were consistent with research on human Septin14 (Peterson et al., 2007). The CDC_septin includes a polybasic domain and a GTP-binding domain of the P-loop superfamily of GTPase, which is a common feature in all septin proteins. The coiled-coil domain is well characterized as a potential protein-protein interaction motif with various complexes. It performs diverse functions, such as helping proteins incorporate into Septin rings and developing normal morphology of Septin rings. Similarly, coiled coils in pig Septin14 may influence Septin-Septin or Septin-membrane interactions.

Subcellular distribution is related to the biological role of a protein, and thus it was used to analyze the role of the pig Septin14. The GFP fluorescence was present in the entire cell in pEGFP-Septin14 plasmid transfected PK15. The localization of Septin14 protein has previously been studied experimentally through transfection assays. Peterson’s group reported that overexpressed GFP-Septin14 (human) localizes to stress fibers in CHO cells, not in the nucleus (Peterson et al., 2007). In the present study, based on the prediction by PSORT software, Septin14 was mainly expressed in the cytoplasm with 47.8% reliability and in the nucleus with 26.1% confidence. The conflict between these results is worth further study.

Multi-tissue expression indicated that the Septin14 gene was almost completely restricted to the testis and seminal vesicle, which is not completely consistent with results from human and mouse (Sugino et al., 2008). Human Septin14 has been reported to be specifically expressed in testis, based on northern blotting and RT-PCR analyses (Peterson et al., 2007). Mouse Septin14 was expressed in developing central nervous tissues (brain) and testis based on immunoblotting, RT-PCR, immunohistochemistry, and immunocytochemistry (Shinoda et al., 2010). The seminal vesicle was not included in the human and mouse studies. Brain was included in the pig and human studies. The expression of Septin14 gene in pig testis is as robust as in human and mouse. The differences in the expression of Septin14 in different tissues or species, such as pigs, humans and mice may be related to inherent properties of Septin14 gene expression. The testis is the main male reproductive gland in all animals, including humans, and produces both sperm and androgens. The seminal vesicles secrete the special significant proportion of the fluid that can become seminal fluid, which dilutes and provides nutrients for the sperm. According to the results presented above, we can infer that pig Septin14 gene participates in sperm functions.

We found that Septin14 mRNA was only minimally expressed at D2, but was highly expressed by D75, when round spermatids became evident in the cross sections of seminiferous tubules in the BMI testes. This suggests that these germ cells are the primary source for Septin14 transcript. However, the Septin14 transcript level is maintained without a major increase between M3 to M15, even though boars reached sexual maturity at M4. It is thus possible that sexual maturity in BMI is less dependent on this protein. Septin14 mRNA was significantly decreased to a low level at Y3, which means its transcript was downregulated in the aged testis. These findings suggested that pig Septin14 is a developmental stage-specific and tissue-specific gene, and thus may function in spermatogenesis. We speculate that Septin14 may function in the division, development and/or apoptosis of germ cells.

In order to obtain Septin14 protein for further functional analysis, we cloned the fusion sequence of Septin14 with pET32a(+), and successfully expressed it in *E.coli* and purified His6-tagged Spetin14 protein. This purified Septin14 protein provides a basis for more detailed studies on its biological functions.

## Conclusion

In this study, we reported pig Septin14 gene for the first time. The full-length cDNA sequence of Septin14 in Banna minipig inbred line (BMI) was cloned using RACE and RT-PCR technologies. Septin14 CDS is 2,514 bp long with a 1,299 bp ORF encoding 432 amino acids, and contains two conserved structural domains: CDC_septin domain and C-terminal coiled coil domain. The mRNA expression profile in 34 pig tissues revealed that Septin14 gene is mainly expressed in testis and seminal vesicle gland. After comparing Septin14 expression at multiple developmental stages, we found that Septin14 mRNA was barely detectable on day 2, but reached the peak level on day 75. This result suggested that Septin14 is a developmental stage-specific and tissue-specific gene, and thus may function in spermatogenesis. Subcellular localization of GFP-tagged Septin14 fusion protein revealed that Septin14 was distributed throughout the testicular cells. Finally, we successfully expressed Spetin14 protein in *E.coli* using a pET prokaryotic expression system and obtained a 67.9 kDa recombinant protein. This study laid the foundation for analyzing the function of pig Septin14 and further studies to elucidate the biological significance of Septin14.
